# Semantic text mining support for lignocellulose research

**DOI:** 10.1186/1472-6947-12-S1-S5

**Published:** 2012-04-30

**Authors:** Marie-Jean Meurs, Caitlin Murphy, Ingo Morgenstern, Greg Butler, Justin Powlowski, Adrian Tsang, René Witte

**Affiliations:** 1 Department of Computer Science and Software Engineering, Concordia University, Montréal, QC, Canada; 2 Centre for Structural and Functional Genomics, Concordia University, Montréal, QC, Canada; 3 Department of Biology, Concordia University, Montreal, QC, Canada; 4 Department of Chemistry and Biochemistry, Concordia University, Montréal, QC, Canada

## Abstract

**Background:**

Biofuels produced from biomass are considered to be promising sustainable alternatives to fossil fuels. The conversion of lignocellulose into fermentable sugars for biofuels production requires the use of enzyme cocktails that can efficiently and economically hydrolyze lignocellulosic biomass. As many fungi naturally break down lignocellulose, the identification and characterization of the enzymes involved is a key challenge in the research and development of biomass-derived products and fuels. One approach to meeting this challenge is to mine the rapidly-expanding repertoire of microbial genomes for enzymes with the appropriate catalytic properties.

**Results:**

Semantic technologies, including natural language processing, ontologies, semantic Web services and Web-based collaboration tools, promise to support users in handling complex data, thereby facilitating knowledge-intensive tasks. An ongoing challenge is to select the appropriate technologies and combine them in a coherent system that brings measurable improvements to the users. We present our ongoing development of a semantic infrastructure in support of genomics-based lignocellulose research. Part of this effort is the automated curation of knowledge from information on fungal enzymes that is available in the literature and genome resources.

**Conclusions:**

Working closely with fungal biology researchers who manually curate the existing literature, we developed ontological natural language processing pipelines integrated in a Web-based interface to assist them in two main tasks: mining the literature for relevant knowledge, and at the same time providing rich and semantically linked information.

## Background

### Introduction

Since the early decades of the 20th century, when the internal combustion engine rapidly replaced the steam engine, transport has been almost totally dependent on fossil fuels. As the petroleum reserves decrease, producing sustainable liquid fuels with low environmental impact is one of the major technological challenges the world is facing today. Industrialized and developing countries consider *biofuels*, fuels produced from biomass, as a promising alternative to fossil fuels.

There are many advantages of using biofuels in terms of economic, environmental and energy security impacts [1]: from biomass sources, biofuels can be sustainable and contribute to reducing carbon dioxide emissions. In the United States, biofuel is produced mainly from the fermentation of hydrolyzed corn starch, a process requiring substantial input of water, fertilizer and energy, and which consumes a food resource. According to the United Nations Environment Programme [2], the global use of biofuels will nearly double during the next ten years. Hence, improving efficiency and sustainability of biofuels production from non-food sources is of great interest. Underutilized agricultural and forestry residues, such as agricultural straws, residues from pulp and paper production and other "green" garbage, are composed of lignocellulose, which is the most abundant organic material on earth.

The conversion of lignocellulose into fermentable sugars for biofuels production requires the use of cocktails of biological catalysts, called enzymes. A key challenge lies in the development of enzyme cocktails that can efficiently and economically hydrolyze lignocellulosic biomass. One approach to meeting this challenge is to mine the rapidly-expanding repertoire of microbial genomes for enzymes with the appropriate catalytic properties [3].

Researchers who aim to identify, analyze and develop these enzymes need to extract and interpret valuable and relevant knowledge from the huge number of documents that are available in multiple, ever-growing repositories.

The largest knowledge source available to biological researchers is the PubMed bibliographic database [4], provided by the US National Center for Biotechnology Information (NCBI), which contains more than 19 million citations from more than 21000 life science journals. PubMed is linked to other databases, like *Entrez Genome*, which provides access to genomic sequences, and *BRENDA, The Comprehensive Enzyme Information System *[5], which is the main collection of enzyme functional data available to the scientific community. A biology researcher querying PubMed using keywords typically collects a long list of potentially relevant papers. Reading all the abstracts and full-text of these papers to extract relevant information is a time-consuming task.

The work we present in this paper focuses on the automatic extraction of knowledge from the massive amount of information on fungal biomass-degrading enzymes available from the literature. In our approach, Natural Language Processing (NLP) pipelines brokered through Web services support the extraction of relevant mentions. Detected entities are further enriched with additional information and where possible, linked to external data sources.

### Related work

To address the challenges of extracting relevant data from large collections of published papers, NLP and Semantic Web approaches are increasingly adopted in biomedical research [6-8]. During the last decade, several systems combining text mining and semantic processing have been developed to help life sciences researchers in extracting knowledge from the literature. Textpresso [9] enables the user to search for categories of biological concepts and classes relating two objects and/or keywords within an entire literature set. GoPubMed [10] supports the arrangement of the abstracts returned from a PubMed query. iHOP [11] converts the information in PubMed into one navigable resource by using genes and proteins as hyperlinks between sentences and abstracts. BioRAT [12] extracts biological information from full-length papers. Bio-Jigsaw [13] is a visual analytics system highlighting connections between biological entities or concepts grounded in the biomedical literature. MutationMiner [14] automates the extraction of mutations and textual annotations describing the impacts of mutations on protein properties from full-text scientific literature. Finally, Reflect [15] is a Firefox plugin which tags gene, protein and small molecule names in any Web page.

### Implementation

Before we describe our overall architecture and the text mining pipelines, we briefly introduce the user groups involved, the semantic entities we analyze and the resources we use.

### System application context

#### User groups

The identification and the development of effective fungal enzyme cocktails are key elements of the biorefinery industry. In this context, the manual curation of fungal genes encoding lignocellulose-active enzymes provides the thorough knowledge necessary to facilitate research and experiments. Researchers involved in this curation are building sharable resources, usually by populating dedicated databases containing the extracted knowledge from the curated literature.

The users of our system are populating and using the mycoCLAP database http://cubique.fungalgenomics.ca/mycoCLAP/[16], which is a searchable database of fungal genes encoding lignocellulose-active proteins that have been biochemically characterized. The *curators *are therefore the first user group of our system. The *biology researchers *who make decisions about the experiments to conduct and the *experimenters *executing them represent two additional user groups. They are mainly interested in the ability of combining multiple semantic queries to the curated data, thereby semantically integrating the various knowledge resources.

#### Semantic entities

The system we are developing has to support the manual curation process; therefore, the semantic entities have been defined by the curators according to the information they need to store in the mycoCLAP database.

Entities include information that is of particular interest for the researchers, such as organisms, enzymes, assays, genes, catalytic properties, substrates, and protein properties. The list of the semantic entities along with the level on which they apply (sentence or word level), their definition and an instance example is provided in Table 1.

**Table 1:** Semantic entities, applicable level (sentence, S or word(s), W), definitions and examples

Semantic entity	Level	Definition
ActivityAssayConditions	*S*	Conditions at which the activity assay is carried out *Ex.: disodium hydrogen phosphate, citric acid, pH 4.0, 37°C*
Assay	*W*	Name of the activity assay *Ex.: Dinitrosalicylic Acid Method (Somogyi-Nelson)*
Enzyme	*W*	Enzyme name *Ex.: alpha-galactosidase*
Gene	*W*	Gene name *Ex.: mel36F*
Glycosylation	*S*	Presence of glycosylation on protein *Ex.: N-glycosylated*
Host	*W*	Organism used to produce the recombinant protein *Ex.: Escherichia coli*
KineticAssayConditions	*S*	Buffer, pH, temp. for the kinetic parameters determination *Ex.: 0.1 M (disodium hydrogen phosphate, citric acid), pH 4.0, 37°C*
Organism	*W*	Organism name *Ex.: Gibberella sp*.
pH	*S*	pH mentions *Ex.: The enzyme retained greater than 90% of its original activity between pH 2.0 and 7.0 at room temperature for 3 h*.
ProductAnalysis	*S*	Products formed from enzyme reaction and identification method *Ex.: HPLC, glucose, galactose*
SpecificActivity	*S*	Specific activity of the enzyme *Ex.: 11.9 U/mg*
Strain	*W*	Strain name *Ex.: F75*
Substrate	*W*	Substrate name *Ex.: stachyose*
SubstrateSpecificity	*S*	Substrate specificity mentions *Ex.: The Endoglucanase from Pyrococcus furiosus had highest activity on cellopentaose*
Temperature	*S*	Temperature mentions *Ex.: The enzyme stability at different pH values was measured by the residual activity after the enzyme was incubated at 25°C for 3 h*.

The list of the semantic entities along with the level they apply (sentence or word level), their definition and an instance example is provided in Table 1.

About half of these entities are detected at the word level (e.g., enzyme or organism names) and the other half consists of contextual properties captured at the sentence level (e.g., pH and temperature contexts). The entity set was built in the perspective of providing instances of the ontological representation of the domain knowledge. The enzyme names are sought, as well as the names of their source organisms and strain designations. The enzymes have specific biochemical properties, such as optimal temperature and pH, temperature and pH stability, specific activity, substrate specificities and kinetic parameters. These experimentally determined properties describe each enzyme's catalytic ability and capacity, and are a basis for comparison between enzymes. Their mentions are captured from the literature along with the laboratory methods (assay) used and the experimental conditions (activity and kinetic assay conditions). In addition to these properties, the extraction of mentions describing an enzymatic property (glycosylation state) and the products formed (product analysis) is performed to complete the knowledge of the reaction.

#### Semantic resources

In terms of knowledge sources, the system relies on external and internal resources and ontologies. The *Taxonomy database *http://www.ncbi.nlm.nih.gov/Taxonomy/[17] from NCBI is used for initializing the NLP resources supporting organism recognition. BRENDA http://www.brenda-enzymes.org[5] provides the enzyme knowledge along with SwissProt/UniProtKB http://www.uniprot.org/[18]. References to the original sources are integrated into the curated data, which allows us to automatically create links using standard Web techniques: e.g., links from an organism mention in a research paper to its corresponding entry in the NCBI Taxonomy database or from an enzyme name to its EC number in BRENDA.

### System design

In this section, we provide an overview of our system architecture, the semantic resources we deployed, and the text mining pipelines we developed.

#### System architecture

With the different user groups and their diverging requirements, as well as the existing and continuously updated project infrastructure, we needed to find solutions for incrementally adding semantic support without disrupting day-to-day work. Our solution deploys a loosely-coupled, service-oriented architecture that provides semantic services through existing and new clients.

**Figure 1:**
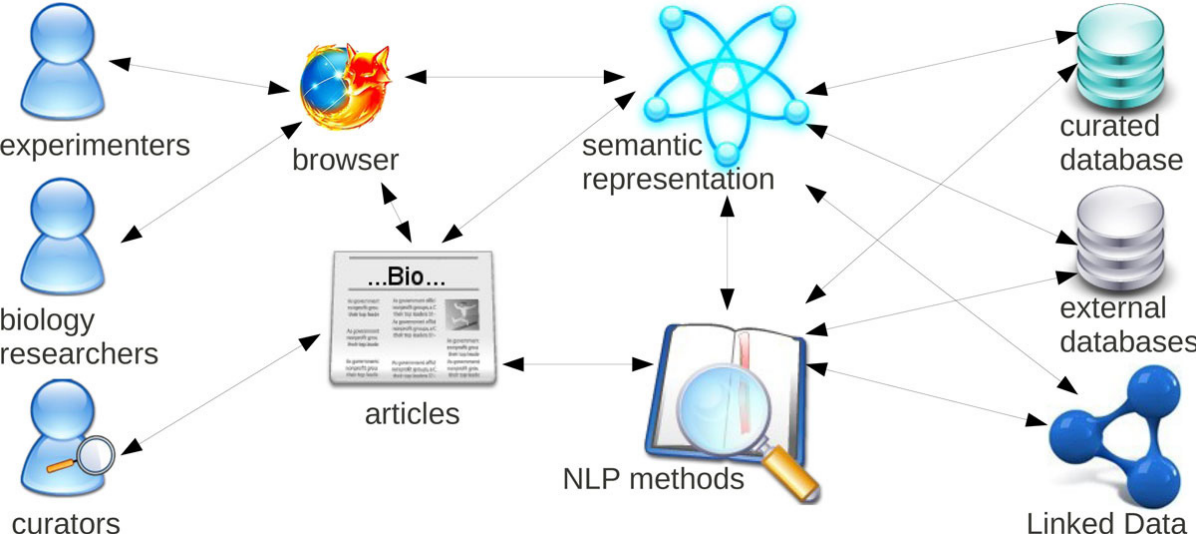
**Integrating semantic support in curation, analysis, and retrieval**. The framework of the semantic support integration in the curation, analysis and retrieval process.

To connect the individual services and their results, we rely on standard semantic data formats, like OWL and RDF, which provide both loose coupling and semantic integration, as new data can be browsed and queried as soon as it is added to the framework (depicted in Figure 1 - Integrating semantic support in curation, analysis, and retrieval). The use of the Semantic Assistants architecture [19] allows us to provide semantic analysis services directly within desktop applications, by leveraging standard SOAP Web services and OWL service descriptions.

#### Ontology

**Figure 2:**
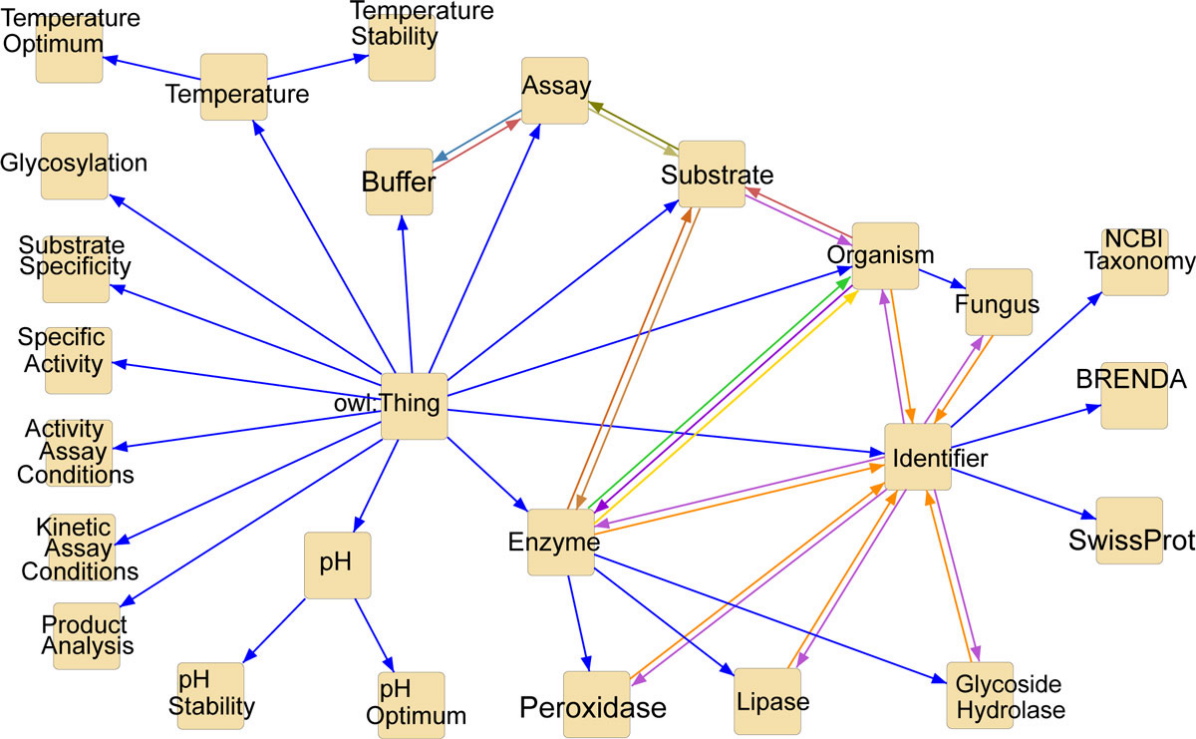
**Domain ontology**. Main entities of the domain ontology and their relationships.

To facilitate semantic discovery, linking and querying the domain concepts across literature and databases, the entities are modeled in an OWL ontology, which is automatically populated from documents. The system presented in this article makes use of the ontology partially depicted in Figure 2 - Domain ontology. The graph nodes show the main entities and the blue arrows represent the subclass relationships, whereas all the other arrows stand for property relationships. The ontology is used both during the text mining process and for querying the extracted information [14].

#### Text mining pipelines

**Figure 3:**
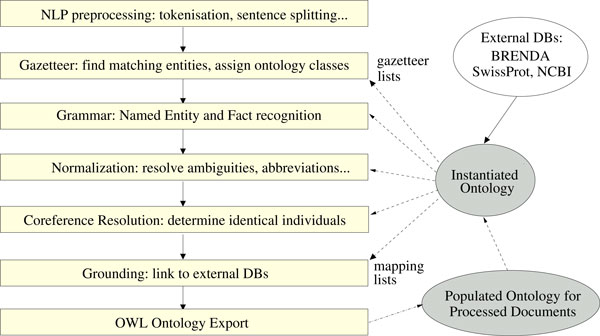
**Natural language processing workflow**. The general workflow of the NLP pipeline.

Our text mining pipelines are based on the *General Architecture for Text Engineering *(GATE) [20]. All documents first undergo basic preprocessing steps using off-the-shelf GATE components. Custom pipelines then extract the semantic entities mentioned above and populate the OWL ontology using the OwlExporter [21] component. The same pipeline can be run for automatic (batch) ontology population, embedded in Teamware (described below) for manual annotation, or brokered to desktop clients through Web services for literature mining and database curation. The general workflow of the pipeline is depicted in Figure 3 - Natural language processing workflow.

##### Preprocessing

The processing resources (PRs) composing the first part of the system pipeline are generic and independent from the domain. Some of these resources are based on standard components shipped with the GATE distribution. In particular, the JAPE language allows the generation of finite-state language transducers that are processing annotation graphs over documents. After initializing the document, the *LigatureFinder PR *finds and replaces all ligatures, like *fi, ff *or *fl*, with their individual characters, thereby facilitating gazetteer-based analysis. The next PR is the *ANNIE English Tokenizer*, which splits the text into very simple tokens, such as numbers, punctuation characters and words of different types. Finally, the *ANNIE Sentence Splitter *segments the text into sentences by means of a cascade of finite-state transducers and the ANNIE part-of-speech (POS) tagger that is included with GATE adds POS tags to each token.

##### Organism recognition

Organism tagging and extraction rely on the open-source OrganismTagger system http://www.semanticsoftware.info/organism-tagger. The OrganismTagger is a hybrid rule-based/machine-learning system that extracts organism mentions from the biomedical literature, normalizes them to their scientific name, and provides grounding to the NCBI Taxonomy database [22].

The OrganismTagger also comes in the form of GATE pipeline, which can be easily integrated into our system. It reuses the NCBI Taxonomy database, which is automatically transformed into NLP resources, thereby ensuring the system stays up-to-date with the NCBI database. The OrganismTagger pipeline provides the flexibility of annotating the species of particular interest to bio-researchers on different corpora, by optionally including detection of common names, acronyms, and strains.

##### Enzyme recognition

Despite the standards published by the Enzyme Commission [23], enzymes are often described by the authors under various formats, ranging from their 'Recommended Name' to different synonyms or abbreviations. Our enzyme recognition process is rule-based: Gazetteer and mapping lists are automatically extracted from the BRENDA database, in addition to a mapping list of SwissProt identifiers extracted from the SwissProt database.

An enzyme-specific text tokenization, along with grammar rules written in the JAPE language, analyses tokens with the -*ase *and -*ases *enzyme suffixes. The gazetteers allow the finding of the enzyme mentions in the documents by applying a pattern-matching approach.

Some abbreviated forms of enzyme names are not found during the pattern matching step, usually because these forms are created by the authors. The following sentence shows an example excerpted from [24].

*The extracellular endoglucanase (EG) was purified to homogeneity from the culture supernatant by ethanol precipitation (75%, v/v), CM Bio-Gel A column chromatography, and Bio-Gel A-0.5 m gel filtration. The purified EG (specific activity 43.33 U/mg protein) was a monomeric protein with a molecular weight of 27 000*.

Here, EG stands for 'endoglucanase', but this abbreviation is not reported in BRENDA. Such abbreviations are meaningful only within the context of a single document. Therefore, our pipeline contains grammar rules identifying these author-specific abbreviations and performing coreference resolution on each document.

The mapping lists link up the enzyme mentions found in the document and the external resources. Through this grounding step, the system provides the user with the enzymes' *Recommended Names, Systematic Names, EC Numbers, SwissProt Identifiers *and the *URL *of the related Web pages on the BRENDA website.

##### Temperature and pH contexts

Temperature and pH mentions are involved in several biochemical contexts, like the temperature and pH dependence/stability of the enzyme, or the description of the activity and kinetic assay conditions. Examples are given in the following sentences from [24]:

**Temperature: ***The purified enzyme exhibited maximum activity at 55°C, with 84% relative activity at 60°C and 29% activity at 70°C under the assay conditions used*.

**pH: ***The enzyme displayed an optimum activity at pH 5.0 and retained 80% activity at pH 3.0 and also at pH 8.0*.

Our GATE pipeline contains PRs based on JAPE rules and gazetteer lists of specific vocabulary that enable the detection of these key mentions at the sentence level.

##### Other entities

The detection of the other entities mentioned in Table 1 is currently implemented through gazetteer lists and grammar rules implemented in JAPE; with the exception of the strain mentions, which are detected by the strain feature provided by the OrganismTagger pipeline.

#### System output and user interfaces

**Figure 4:**
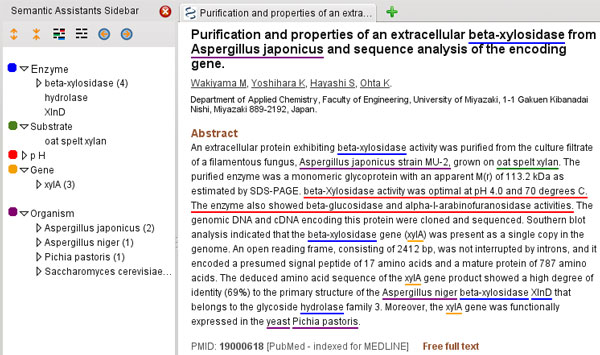
**Text mining results displayed in Firefox through the Semantic Assistants plug-in**. Text mining results are displayed in Firefox through the Semantic Assistants plug-in.

The system output supports two different tasks: the manual annotation of reference papers needed for evaluation purposes and the database curation manually performed by the biologists. In the context of manual annotation, the original papers are enriched with the system output added as pre-annotations before being submitted to the human annotators. In the context of database curation, all text mining pipelines are brokered as NLP Web services through the Semantic Assistants framework [19]. Users can access these services from their desktop through client plug-ins for common tools, such as the Firefox web browser (Figure 4 - Text mining results displayed in Firefox through the Semantic Assistants plug-in) or the OpenOffice word processor. This provides the biologists using our system with the ability to quickly invoke semantic analysis services on scientific documents they browse online or edit in their text processor, without having to switch to an external text mining application.

External resources can be accessed from the user interfaces; the system output provides direct links to the relevant Web pages, e.g., URLs of the Web pages related to the detected enzymes on the BRENDA website site or the detected organisms on the NCBI Taxonomy website.

## Results and discussion

In this section, we first discuss the development of the gold standard corpus and present preliminary results of our system.

### Manual annotation process

For the intrinsic evaluation of our NLP pipelines, we are building a gold standard corpus of freely accessible full-text articles. These are manually annotated through GATE Teamware [25], a Web-based management platform for collaborative annotation and curation.

**Figure 5:**
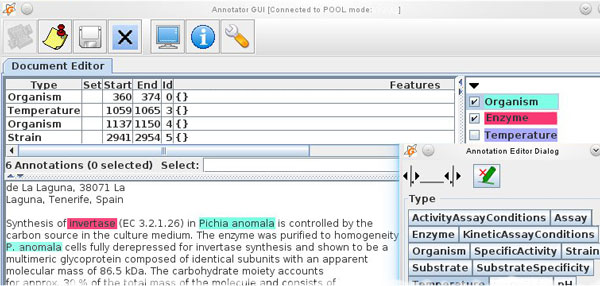
**Teamware annotator GUI**. The annotator's interface in GATE Teamware.

The tool reports on project status, annotator activity and statistics. The annotator's interface (see Figure 5 - Teamware annotator GUI) allows the curator to view, add and edit text annotations that are either manually created using the Teamware interface or pre-annotated. We make use of that ability by providing the annotators with documents we pre-annotate with our NLP pipelines throughout its development.

The annotation team consists of four biology researchers. The researcher in charge of the curation task and an annotator having a strong background in fungal enzyme literature curation are considered as **expert **annotators. The inter-annotator agreement between them is over 80% (F-measure), hence their annotation sets are always defined as the most reliable sets during the adjudication process.

### Corpus

The corpus is composed of freely accessible full-text articles containing critical knowledge and technical details the biology researchers aim to store in the mycoCLAP database which is specifically designed for their needs. The papers are related to classes of enzymes, among them the glycoside hydrolases, the lipases and the peroxidases. Glycoside hydrolase papers represent 69%, lipase papers account for 12% of the articles, and the remaining 19% are related to peroxidases. The current gold standard corpus is composed of ten full-text papers that have been manually annotated by four biologists each.

At the word level, the two most common entities are enzymes and organisms, while the most common at the sentence level are pH and temperature. Table 2 shows these entities and their counts of occurrence in the current gold standard corpus. The goal for the current annotation task is to include fifty manually annotated papers in the gold standard corpus. This corpus will be available on demand.

**Table 2:** Entities and their counts in the current gold standard corpus

Entity	Counts
**Enzyme**	1493
**Organism**	984
**pH**	110
**Temperature**	115

Table 2 shows the most common entities and their counts of occurrence in the current gold standard corpus.

### Results

The performance of our text mining pipelines is evaluated in terms of precision, recall and F-measure. Here, the reference is provided by the gold standard corpus. Precision is defined as the number of correct tags detected by the system divided by the total number of detected tags. Recall is defined as the number of correct tags detected by the system divided by the total number of reference tags. The F-measure is the harmonic mean of precision and recall. For the 'strict' evaluation, we consider all partially correct responses as incorrect, while 'lenient' considers all partially correct (overlapping) responses as correct. In this evaluation, we focus on the four most common entities (Enzyme, Organism, pH and Temperature) in our currently annotated corpus. The results of the text mining pipelines are shown in Table 3.

**Table 3:** Text Mining pipelines results on the gold standard corpus in terms of recall (R), precision (P) and F-measure (Fm)

	Strict			Lenient		
	R	P	Fm	R	P	Fm
**Enzyme**	0.79	0.64	0.71	0.91	0.75	0.82
**Organism**	0.87	0.86	0.87	0.91	0.91	0.91
**pH**	0.79	0.81	0.80	0.96	0.99	0.98
**Temperature**	0.70	0.66	0.68	0.93	0.88	0.91

The results of the text mining pipelines on the four most common entities (Enzyme, Organism, pH and Temperature) in terms of recall (R), precision (P) and F-measure (Fm) are shown in Table 3.

## Discussion

The OrganismTagger performance has previously been evaluated on two corpora, where it showed a precision of 95%-99%, a recall of 94%-97%, and a grounding accuracy of 97.4%-97.5% [22]. Since its results here are lower, we examined the error cases in more detail.

The manual annotation of organisms highlights all the textual mentions referring to an organism as indirect references, non-standard names (e.g., non-binomial names) or generic mentions. In some cases, correct results from the OrganismTagger were not manually annotated, leading to false positives. The following common sentence:

*Soluble protein was determined according to the method of Lowry et al. (1951) using **bovine **serum albumin as standard*.

shows an example of such a case where the OrganismTagger correctly annotates *bovine *as an organism, whereas the expert annotators considered *bovine serum albumin *as a stand-alone expression.

In some other cases, human annotations are not detected by the OrganismTagger. For example, *Trichoderma viridie *and *M. incrasata *or *cellulolytic fungi* were manually annotated as organisms by the experts. These mentions are not detected by the OrganismTagger. In the first two cases, the cause is a spelling difference between the names of the organisms reported in the NCBI Taxonomy database and their mention in the article. In the last case, the annotation of a generic organism mention that is relevant within the context of our project is not an objective of the OrganismTagger system, which is designed to provide normalization with scientific names and grounding to the NCBI Taxonomy database. Consequently, the results obtained by our pipeline on the organism recognition are lower than the published results of the OrganismTagger system. The text mining pipeline supporting our system needs to be enhanced in its ability to capture generic organism mentions and to discard stand-alone expressions containing organism names.

The results obtained on *Temperature *and *pH *sentence detection are much better in the lenient evaluation than the strict because of sentence splitter mistakes.

The enzyme recognition pipeline provides state-of-the-art performance. However, wrong detection of abbreviations and acronyms represent 92% of the false negatives found by our pipeline. Further work is needed to reduce this amount by improving the co-reference resolution with approaches as described in [26] and external resources, such as Allie [27].

## Conclusions

We presented our ongoing development of a semantic infrastructure for enzyme data management. As the first system specifically designed for lignocellulolytic enzymes research, it targets the automatic extraction of knowledge on fungal enzymes from the research literature. The proposed approach is based on text mining pipelines combined with ontological resources. Preliminary experiments show state-of-the-art results. Improving the consistency of the extracted knowledge by increasing the use of ontologies is one of the next goals for our system. Therefore, a key objective is the population of the overall ontology of the domain knowledge and its publication in Linked Data format.

The gold standard corpus of manually annotated papers, as well as the presented system, will be available under http://www.semanticsoftware.info/genozymes.

The accessibility of the services through the Semantic Assistants framework allows the users to mine the semantically annotated literature from their desktop. Future work is needed to enable the interaction between selected users (e.g., curators) and the presented system in terms of data validation and knowledge acquisition.

In future work, we will further deploy our text mining pipelines to assess the quality of existing manually curated data in the databases. Measuring the overall impact of the semantic system on the scientific discovery workflow will be the target of an extrinsic study.

## List of abbreviations

ANNIE: a Nearly-New Information Extraction System; BRENDA: BRaunschweig ENzyme DAtabase; EC: Enzyme Commission; GATE: General Architecture for Text Engineering; GUI: Graphical User Interface; JAPE: Java Annotation Patterns Engine; mycoCLAP: (database of) Characterized Lignocellulose-Active Proteins of fungal origin; NCBI: National Center for Biotechnology Information; NLP: Natural Language Processing; OWL: Web Ontology Language; POS: Part Of Speech; PR: Processing Resource; RDF: Resource Description Framework; SOAP: Simple Object Access Protocol; URL: Uniform Resource Locator.

## Competing interests

The authors declare that they have no competing interests.

## Authors' contributions

MJM implemented the system, carried out the ontology, provided GATE and NLP expertise, participated in curation and evaluation and drafted the manuscript. CM carried out the curation, participated in the ontology design and the system evaluation. IM carried out the curation, participated in the ontology design and the system evaluation. GB participated in the concept and approach definitions the study makes use of and participated in the fungal genomics application. JP performed the curation validation, provided expertise on fungal enzymes and their literature and reviewed the manuscript. AT conceived of the study, participated in its design and the fungal genomics application, provided overall direction of the project and reviewed the manuscript. RW participated in the concept and approach definitions the study makes use of, provided GATE and NLP expertise, contributed to the Semantic Assistants framework, and reviewed the manuscript. All authors read and approved the final manuscript.

## Authors' information

MJM is a postdoctoral fellow, a member of the Semantic Software Lab and the Centre for Structural and Functional Genomics at Concordia University. CM is a research associate at the Centre for Structural and Functional Genomics at Concordia University. IM is a postdoctoral fellow and a member of the Centre for Structural and Functional Genomics at Concordia University. GB is a professor in computer science and a member of the Centre for Structural and Functional Genomics at Concordia University. He leads the bioinformatics group. JP is an associate professor in biochemistry and a member of the Centre for Structural and Functional Genomics at Concordia University. AT is a professor of biology and the director of the Centre for Structural and Functional Genomics at Concordia University. He is the principal investigator of the project that supports this work. RW is an assistant professor in computer science and software engineering at Concordia University. He is the leader of the Semantic Software Lab.
